# Identification of Tetranectin as a Potential Biomarker for Metastatic Oral Cancer

**DOI:** 10.3390/ijms11093106

**Published:** 2010-09-02

**Authors:** Martha E. Arellano-Garcia, Roger Li, Xiaojun Liu, Yongming Xie, Xiaofei Yan, Joseph A. Loo, Shen Hu

**Affiliations:** 1 School of Dentistry and Dental Research Institute, University of California Los Angeles, Los Angeles, CA, USA; E-Mails: martha_eliz6@yahoo.com (M.E.A.-G.); liuliuxj@ucla.edu (X.L.); 2 Department of Chemistry and Biochemistry, University of California Los Angeles, Los Angeles, CA, USA; E-Mails: rl8402@gmail.com (R.L.); yongming.xie@bruker.com.cn (Y.X.); jloo@chem.ucla.edu (J.A.L.); 3 Department of Statistics, University of California Los Angeles, Los Angeles, CA, USA; E-Mail: yanxiaofei54@gmail.com; 4 Department of Biological Chemistry, David Geffen School of Medicine, University of California Los Angeles, Los Angeles, CA, USA; 5 Jonsson Comprehensive Cancer Center, University of California Los Angeles, Los Angeles, CA, USA

**Keywords:** oral squamous cell carcinoma, serum proteomics, tetranectin, disease biomarker

## Abstract

Lymph node involvement is the most important predictor of survival rates in patients with oral squamous cell carcinoma (OSCC). A biomarker that can indicate lymph node metastasis would be valuable to classify patients with OSCC for optimal treatment. In this study, we have performed a serum proteomic analysis of OSCC using 2-D gel electrophoresis and liquid chromatography/tandem mass spectrometry. One of the down-regulated proteins in OSCC was identified as tetranectin, which is a protein encoded by the *CLEC3B* gene (C-type lectin domain family 3, member B). We further tested the protein level in serum and saliva from patients with lymph-node metastatic and primary OSCC. Tetranectin was found significantly under-expressed in both serum and saliva of metastatic OSCC compared to primary OSCC. Our results suggest that serum or saliva tetranectin may serve as a potential biomarker for metastatic OSCC. Other candidate serum biomarkers for OSCC included superoxide dismutase, ficolin 2, CD-5 antigen-like protein, RalA binding protein 1, plasma retinol-binding protein and transthyretin. Their clinical utility for OSCC detection remains to be further tested in cancer patients.

## 1. Introduction

Oral cancer, predominantly oral squamous cell carcinoma (OSCC), is the sixth most common human cancer with an annual incidence of over 300,000 new cases worldwide [1,2]. The disease frequently metastasizes to the lymph nodes, which represents the most important predictor of patient survival rates. Typically, 50% of patients with OSCC have detectable lymph node involvement at presentation. Less than 40% of patients with lymph node metastasis at presentation survive five years, compared to ~90% of patients without metastasis [3,4,5]. In other words, the survival rate decreases by approximately 50% when nodal metastasis is present. Treatment of individuals clinically diagnosed with lymph node metastasis (N+) often involves a surgical procedure of radical neck dissection (RND) to remove a substantial portion of the neck. Upon histological examination of removed tissues, 10–20% of clinically diagnosed N+ individuals turn out to be metastasis-free (N0) [6]. Clinical diagnosis of N0 status is even less accurate. Postoperative histological examination shows that approximately one-third of clinically diagnosed N0 individuals have metastasis-positive lymph nodes in the neck [7]. In fact, due to the low sensitivity of clinically detecting nodal metastasis and the poor prognosis when these metastases are missed, the current management of the clinically diagnosed N0 neck commonly includes routine elective neck dissection (END) with pathologic examination of the removed lymph nodes [8]. END is less appropriate than RND for N+ individuals falsely diagnosed as N0 and, moreover, is completely unnecessary for individuals correctly diagnosed as N0. Although END is less rigorous than RND, the treatment causes disfigurement, long-term discomfort and pain and can lead to additional complications such as shoulder and neck disability [9].

To ensure optimal management of OSCC patients, there is a critical need for accurate staging of lymph nodes metastasis in the neck. Unfortunately, current preoperative clinical methods, including new radiographic techniques, are suboptimal and misdiagnose the presence or absence of cervical nodal metastasis in many patients [8]. Cancer biomarkers may be useful for prediction/detection of lymph-node metastasis in patients with OSCC. These biomarkers may help differentiate patients who clinically have no detectable disease but are potential candidates for lymph nodes metastasis and should have neck dissection and/or adjuvant radiotherapy. Conversely, the biomarkers would also help avoid unnecessary surgery treatment for metastasis-free patients. This notion was demonstrated in a quantitative PCR study to screen 40 potential markers for their ability to detect head and neck SCC (HNSCC) metastases to cervical lymph nodes. Four markers were able to discriminate between histologically positive and benign nodes with accuracy >97% and one marker, pemphigus vulgaris antigen, provided discrimination with 100% accuracy and correctly identified two lymph nodes with micrometastatic tumor deposits [10]. Many previous studies have also revealed proteins that promote the metastasis in oral/head and neck cancer (Table 1). Further validation of these proteins in metastatic and primary OSCCs may lead to valuable biomarkers for clinical staging of OSCC.

In this study, we have performed a serum proteomic analysis of OSCC using two-dimensional gel electrophoresis (2-DE) protein separation with liquid chromatography-tandem mass spectrometry (LC-MS/MS) for protein identification. One of the down-regulated proteins in OSCC patients’ sera was identified as tetranectin, which is a ubiquitously expressed lectin protein. This protein was previously found at reduced levels in patients with solid malignant tumors (breast, colon, and cervical cancer) [44–46]. We further measured the levels of tetranectin from patients with metastatic and primary OSCC, and both serum and saliva tetranectin was found significantly reduced in patients with metastatic OSCC.

## 2. Materials and methods

### 2.1. Patients and Samples

In total, 34 OSCC and 10 healthy subjects were used in this study. Sixteen cancer patients were at early stage without lymph node metastasis (N = 0) whereas the other 18 patients had lymph-node metastasis (N+). All patients provided consent according to the institutional review board of University of California, Los Angeles. Patients did not receive any treatment prior to serum or saliva collection. Standardized protocol was used for collection, storage, and processing of the serum and saliva samples, as described previously [47,48]. The samples were aliquoted and stored at −80 °C.

### 2.2. 2-D Gel Electrophoresis

Serum samples from 10 OSCCs and 10 matched control subjects were used for 2-DE analysis. There were no significant differences in terms of mean age, gender or smoking history (p > 0.75). All the serum samples were precipitated by rapid adding four times volume of cold acetone containing 10% (w/v) trichloroacetic acid (TCA). The mixture was kept at −20 °C overnight and centrifuged at 13,200 g, 4 °C, for 15 minutes. The pellet was then washed with cold acetone followed by centrifugation at 13,200 g, 4 °C for 15 minutes. The sample pretreatment step helped remove most of albumin from serum/plasma samples [49]. After removing the supernatant, the pellet was dissolved in rehydration buffer and stored at −80 °C prior to 2-DE analysis. Protein assay was performed using the 2-D Quant Kit (Amersham).

A total of 20 protein samples (10 OSCC and 10 control, 150 μg proteins each sample) were individually mapped out using 2-DE. Isoelectric focusing (IEF) was performed using the immobilized pH gradient (IPG) strips (17-cm length, p*I* 3–10 NL) on a Protean IEF cell, and SDS-PAGE was performed with the 8–16% precast Protean II gels (Bio-Rad). Proteins were visualized with the fluorescent Sypro-Ruby staining (Molecular Probes).

Analysis of the 2-D gel images was performed using the PDQuest (Bio-Rad). A matchset was created initially, and protein spots were automatically matched and further verified. Afterwards, normalization was performed based on the total density of the gel image and the levels of proteins were quantified.

### 2.3. LC-MS/MS and Database Searching

Protein spots of interest were excised using a spot-excision robot (Proteome Works, Bio-Rad) and deposited into 96-well plates. Proteins in gel spots were reduced with 10 mM DTT for 30 min, followed by alkylation with 50 mM iodoacetamide for 60 min in the dark, and then digested with 10-ng trypsin at 37 °C overnight. After digestion, all the samples were dried and re-constituted with 0.1% formic acid for subsequent LC-MS/MS analysis.

LC-MS/MS was performed using a LC Packings nano-LC system (Sunnyvale, CA, USA) with a nanoelectrospray interface (Protana, Odense, Denmark) and quadrupole time-of-flight (Q-TOF) mass spectrometer (Applied Biosystems, QSTAR XL, Foster City, CA, USA). A New Objective (Woburn, MA, USA) PicoTip tip (I. D., 8 mm) was used for spraying with the voltage at 1850 V for online MS and MS/MS analyses. Nano-LC separation of peptides was performed with home-made C18 precolumns (300 μm × 1 mm; particle size 5 μm) and LC Packings PepMap C18 columns (75 μm × 150 mm; particle size 5 μm) at a flow rate of 250 nL/min. The eluents used for the LC were (A) 5% ACN/95% H_2_O/0.1% FA and (B) 95% ACN/5% H_2_O/0.1% FA. A gradient was utilized from 5% B to 60% B in 55 min, and ramped to 95% B in 0.1 min. After 5 min at 95% B, the column was re-equilibrated for 15 min before the next run.

Database searching was performed against the EBI human IPI database (version 3.03) using the Mascot database search engine (version 2.1, Matrix Science, London, UK). One missed tryptic cleavage was allowed, and a mass tolerance of 0.3 Da was set for the precursor and product ions. A Mascot score of >40 with a p-value < 0.05 was considered a significant match of a peptide. The MS/MS spectra for each peptide were manually examined to verify the identification.

### 2.4. Western Blot Analysis

Western blotting was used to measure the level of tetranectin in a new set of OSCC serum samples (12 primary/12 metastatic OSCC). Proteins were separated on 12% SDS-PAGE gels at 150 V for about one hour and then transferred to polyvinylidene difluoride membrane using a Mini Trans-Blot electrophoretic transfer cell (Bio-Rad). After saturating with 5% milk in TBST buffer overnight at 4 °C, the blots were sequentially incubated with primary antibody (Lab Vision, 1:250 dilution) and horseradish peroxidase-conjugated antimouse IgG secondary antibody (Amersham, 1:5000 dilution). Finally, the bands were visualized by enhanced chemiluminescence detection (Amersham). Tetranectin was also validated in the saliva samples of 12 primary and 12 metastatic OSCCs. The level of tetranectin was normalized against actin for quantification, and p values were determined using the student’s T-test.

## 3. Results

**Figure 1:**
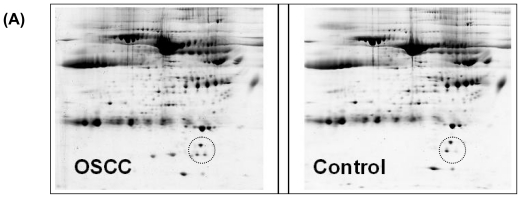

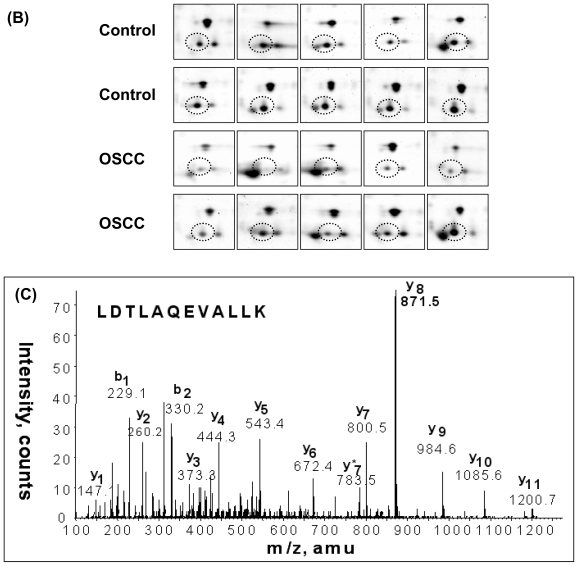
Analysis of serum proteins from OSCC and healthy control subjects using 2-DE with tandem MS. **(A)** 2-DE separation of serum proteins. 1st-dimension separation: IEF, IPG strips (*p*I 3–10, non-linear. 2nd-dimension: SDS-PAGE, 18-cm 8–16% gradient gels. The proteins were visualized using Sypro Ruby staining; **(B)** Serum tetranectin (circled) was found at significantly reduced level in OSCCs (n = 10) compared to healthy controls (n = 10), based on 2-DE analysis; **(C)** ESI-MS/MS spectrum of a tryptic peptide, LDTLAQEVALLK, originated from tetranectin. The protein was identified by using in-gel digestion and LC-MS/MS analysis of the resulting peptides.

Instead of analyzing pooled samples from patient or control groups, we individually mapped out serum proteins from 10 OSCC patient samples and 10 matched controls with 2-DE. Figure 1A shows representative 2-D gel patterns of serum proteins from OSCC and healthy subjects. With initial normalization of the spot intensities and subsequent statistical t-test analysis, 32 protein spots were found with significantly different abundances between the OSCC and control groups (p < 0.05), including 18 up-regulated and 20 down-regulated ones in OSCC. A list of these up- or down-regulated serum proteins identified by LC-MS/MS is shown in Table 2.

**Figure 2:**
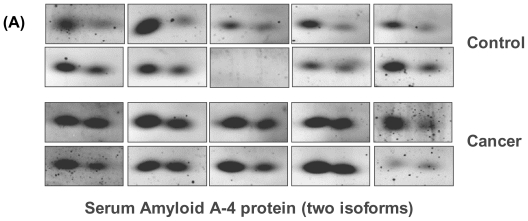

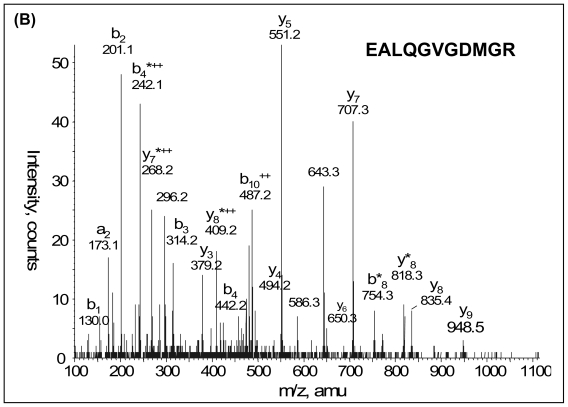
Serum amyloid A-4 protein (SAA-4) is significantly over-expressed in OSCC compared to healthy individuals. **(A)** Both isoforms of SAA-4 were found significantly higher in OSCC than healthy controls, based on 2-DE analysis; **(B)** The ESI-MS/MS spectrum for a tryptic peptide, EALQGVGDMGR, derived from SAA-4.

Figure 1B shows the level of tetranectin among the 10 OSCC and 10 healthy control subjects based on 2-DE analysis. The protein was significantly down-regulated in OSCC patients compared to the healthy control subjects. Figure 1C depicts the tandem MS spectrum for a doubly charged tryptic peptide (m/z 697.4), LDTLAQEVALLK, originated from tetranectin. The precursor ion was well fragmented to yield sufficient structural information to identify the peptide sequence based on Mascot database searching. In total, 8 peptides derived from tetranectin were identified, resulting in an overall sequence coverage of 32%. Conversely, both isoforms of serum amyloid A-4 (SAA-4) were up-regulated in patients with OSCC (Figure 2).

**Figure 3:**
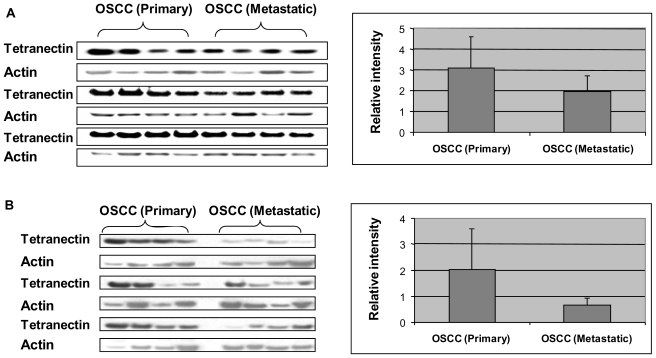
**(A)** Western blot analysis of tetranectin in serum samples from primary (n = 12) and lymph-node metastatic (n = 12) OSCC subjects; **(B)** Western blotting of tetranectin in saliva samples from primary OSCC (n = 12) and lymph-node metastatic OSCC (n = 12). The bar figures show the normalized level (y-axis) of tetranectin against actin. Tetranectin was significantly under-expressed in metastatic *versus* primary cancer (Serum: p = 0.03; saliva: p = 0.007).

Using Western blot analysis, we tested the level of tetranectin in an independent set of primary and metastatic OSCC patients (Figure 3). Tetranectin was found significantly under-expressed in serum (p = 0.03) and saliva (p = 0.007) of metastatic OSCC compared to primary OSCC. Besides, the level of tetranectin was further confirmed by Western blotting on a set of OSCC and control serum samples (n = 25 for each group). The protein level was found to be significantly lower (p = 0.004) in OSCC than in healthy control samples, which agrees to the result obtained by 2-DE.

## 4. Discussion

Our study suggests that tetranectin is a potential biomarker for metastatic OSCC because tetranectin in both serum and saliva is significantly reduced in metastatic OSCC compared to primary OSCC. Tetranectin belongs to a distinct group of the C-type lectin superfamily. In fact, human tetranectin is a homotrimer forming a triple alpha-helical coiled coil. Each monomer consists of a C-type lectin domain (*i.e*., carbohydrate recognition domain) connected to a long alpha helix [50]. Tetranectin binds specifically to the kringle 4 domain of plasminogen, which is a circulating zymogen [51]. For several decades, it has been postulated that plasminogen activation plays an important role in tumor invasion and metastasis. Plasminogen activators released from cancer cells may catalyze the proteolytic conversion of the inactive zymogen plasminogen to the active protease plasmin, which in turn catalyzes degradation of proteins in basement membranes and extracellular matrix (ECM) and thus facilitates cancer cell invasion into the surrounding tissue. There are two types of plasminogen activators (PA), the urokinase-type (uPA) and the tissue-type (tPA). uPA is generally agreed to be the enzyme of most relevance to tumor biology, while the primary role of tPA is generation of plasmin for fibrinolysis in blood vessels. Several other proteins, fibrin, uPA receptor (uPAR), and two main PA inhibitors, PAI-1 and PAI-2, are also important in this process. Fibrin is a cofactor for plasminogen activation by tPA whereas uPAR (cell membrane-anchored uPA binding protein) is a cofactor for plasminogen activation by uPA [52]. Besides plasminogen, tetranectin also specifically binds to the plasminogen-like hepatocyte growth factor and tPA, in a calcium dependent way, but does not interact with uPA. The enhanced activation was suggested to be caused by tetranectin’s ability to bind and accumulate tPA in an active conformation [53]. PAI-1 and PAI-2 belong to the serine protease inhibitor super-family and are involved in the regulation and inhibition of binding between uPA and its receptor. PAI-2 has been identified as a potential biomarker for invasive HNSCC, since PAI-2 expression at both mRNA and protein levels decreased dramatically in cultured HNSCC cells as well as in HNSCC biopsy specimens when invasion into the underlying connective stroma occurred [20].

Tetranectin was initially implicated as a cancer biomarker because decreased plasma levels of tetranectin correlated with cancer progression [46,54]. In the case of ovarian cancer, decreased plasma levels of tetranectin were found as a stronger predictor of adverse prognosis than cancer stage [55]. Low levels of serum tetranectin also correlated with poor treatment responses in patients with metastatic breast cancer [45,46]. These results, including ours about OSCC, corroborate that tetranectin may be used as a marker for metastasis and classification of patients with solid tumor of epithelial origin.

Although the precise biological function of tetranectin in tumorigenesis is unknown, the protein likely has a role in cancer progression by enabling tumour cell invasion and metastasis through activation of the plasminogen-cascade and enhancement of the proteolytic processes. By binding to plasminogen, tetranectin may enhance activation of plasminogen to plasmin, which plays a role in the degradation of extracellular proteins and cancer progression. Tetranectin is present in the stroma of breast, ovary, colon cancers but not present in corresponding normal tissue [56,57]. Based on immunohistochemistry (IHC) studies, tetranectin was found in a fibrillar-like pattern in the ECM around the tumour islands but not detectable in the normal colon stromal tissue. These observations suggest that tetranectin may be produced locally by cells of the stromal compartment of tumours and then deposited into the ECM. In addition, tetranectin exhibited a similar IHC staining pattern as plasminogen in colon cancer and co-localized with plasminogen/plasmin at the invasive front of cutaneous melanoma lesions, indicating a coordinated role of these proteins in the invasive process [57,58]. Positive staining for tetranectin in cancer stroma also strongly correlated with cancer progression [55,59].

The differential level of saliva tetranectin between metastatic and primary OSCC appeared to be more significant than that of serum tetranectin. Oral cancer cells are immersed in the salivary milieu. Tetranectin seems to be consumed in the tumour microenvironment where proteolytic activity is needed for tumour metastasis, and therefore less amount of tetranectin is present in saliva of patients with metastatic tumour than primary tumour. Nevertheless, saliva is an easily accessible body fluid that can be used for survey of the general health and diagnosis of human diseases [60–63]. Salivary testing provides several key advantages including low cost, non-invasiveness, and easy sample collection, especially under circumstances when it is difficult to obtain blood samples. In fact, comparisons between the proteomes of plasma and saliva have indicated that a number of proteins found in saliva are present in blood [64,65]. Testing of protein biomarkers such as tetranectin in saliva from patients with OSCC may be useful for classification of OSCC patients.

## 5. Conclusion

In summary, we have demonstrated that serum proteomics is a valuable approach to discovery of protein biomarkers for oral cancer. Besides tetranectin, this study also revealed candidate biomarkers such as SAA-4, superoxide dismutase, ficolin 2, CD-5 antigen-like protein, plasma retinol-binding protein, RalA binding protein 1 and transthyretin that may have diagnostic value for OSCC. SAA was confirmed as a prognostic biomarker for myeloma in a recent study. SAA combined with C reactive protein might be used as prognostic serological biomarkers in early-stage melanoma patients, helping to discriminate low-risk patients from high-risk patients needing adjuvant treatment [66]. Further studies are needed to enroll additional patients aimed to validate these potential biomarkers including tetranectin. If successfully validated, protein biomarkers such as tetranectin may be used to classify patients with lymph node metastasis for appropriate treatment.

## Figures and Tables

**Table 1:** A partial list of reported proteins promoting metastasis in HNSCC.

Protein	Up/Down	Role in metastasis	Reference
Chemokine receptor 6	Down-regulation	Controls immune cell trafficking in response to inflammatory stimuli	[11]
Endostatin & Collagen XVII	Down-regulation	Angiogenesis inhibitor	[12]
CD44	Down-regulation	Cell adhesion molecule	[13,14]
Lin-7C/VELI3/MALS-3	Down-regulation	Signals through β-catenin	[15]
E-cadherin, β-catenin	Down-regulation	Adhesion molecule, reduction in cell-cell adhesion	[14]
Maspin	Down-regulation	Member of the serpin family of protease inhibitors	[16]
NM23-H1/NDPK	Down-regulation	Metastasis suppressor factor	[17,18]
Nicotinamide N-Methyltransferase (NNMT)	Down-regulation	Enzyme participating in nicotinate and nicotinamide metabolism	[19]
PAI-2	Down-regulation	Inhibits conversion of plasminogen to plasmin and inhibit fibrolysis	[20]
Pemphigus vulgaris antigen	Up-regulation	Cell adhesion molecule	[21]
MMP-1, 2, 3, 9, MT1-MMP, TIMP-1	Up-regulation	Proteolytic activity against the components of ECM, MT1-MMP activates MMP-2, TIMP-1 inhibits MMP-2	[22]
Chemokine receptor 7	Up-regulation	Mediator of immune cell survival and migration to lymph nodes	[11,23]
Cyclin D1	Up-regulation	Cell cycle regulator	[24,25]
Cathepsin B & L	Up-regulation	Lysosomal proteolytic enzymes	[26]
Stefin A and B	Up-regulation	Inhibitors of cathepsin B and L	[26]
HIF-1 alpha	Up-regulation	Promotes tumor progression and metastasis	[27]
TWIST	Up-regulation	Essential mediator of cancer metastasis	[27]
PLC γ-1	Up-regulation	Promotes tumor cell invasion when activated by EGFR	[28,29]
c-Src	Up-regulation	Proto-oncogene	[28]
EGFR & TGF-alpha	Up-regulation	Cell proliferation activator	[30–32]
pSTAT3	Up-regulation	Transcription factor, regulates MMP-2	[24,31]
SDF-1 alpha	Up-regulation	Promotes metastasis through activation of NF-kappa B signaling	[33]
Met	Up-regulation	Increases MMP-9	[34]
NBS1	Up-regulation	Up-regulates Snail and MMP-2	[35]
Integrin beta 6	Up-regulation	Activates Fyn and promotes oral cancer progression	[36]
Cyclin B1	Up-regulation	Cell cycle regulator	[37]
Erythropoietin (EPO) & EPO receptor	Up-regulation	Activates JAK-STAT signaling and cell invasion	[38]
FAK	Up-regulation	Cell differentiation and cell invasion	[39]
HER-2 & HER-3	Up-regulation	Cell growth and differentiation	[32,40]
Integrin alpha v beta 6	Up-regulation	Promotes tumor growth and invasion, activates MMP-3	[41]
VEGF & Interleukin 8	Up-regulation	Promotes tumor angiogenesis, growth and metastasis	[42]
Hepatocyte growth factor (HGF)	Up-regulation	Induces the expression of Interleukin 8 and VEGF	[43]

**Table 2:** A list of identified serum proteins with significantly different abundances between the oral cancer and control subjects.

Accession	Protein Name	Isoelectric Point	Molecular Weight	Peptides Identified	P Value	Ratio (ctrl/cancer)
IPI00332128	48 kD Protein	5	48396	2	0.021	0.28
IPI00384697	ALB protein	5.97	47330	8	0.048	1.72
IPI00022429	Alpha-1-acid glycoprotein 1	5.11	24770	13	0.030	1.99
IPI00020091	Alpha-1-acid glycoprotein 2	5.03	23588	2	0.029	1.59
IPI00305457	Alpha-1-antitrypsin	5.19	48208	12	0.018	0.47
IPI00021842	Apolipoprotein E	5.65	36132	6	0.040	1.85
IPI00177869	Apolipoprotein L1 Isoform A	5.6	43947	2	0.015	1.68
IPI00025204	CD-5 Antigen like protein	5.28	38063	7	0.029	1.59
IPI00164623	Complement C3 fragment	6.02	187046	4	0.022	0.00
IPI00027827	Extracellular superoxide dismutase	6.14	25865	2	0.031	0.27
IPI00017530	Ficolin 2	6.09	33998	2	0.019	0.65
IPI00478493	Haptoglobin	6.13	45177	10	0.041	0.35
IPI00296170	Haptoglobin-related protein	6.42	43049	2	0.003	2.00
IPI00477597	Haptoglobin-related protein	6.42	38983	5	0.012	5.59
IPI00431645	HP protein	8.48	31362	4	0.006	0.55
IPI00472610	Hypothetical protein	7.5	52633	10	0.013	2.32
IPI00332161	Ig gamma 1 Chain C region	8.46	36083	8	0.020	0.41
IPI00385058	Ig kappa chain C region	7.51	26283	4	0.031	1.81
IPI00335356	Ig mu chain C region	6.35	49526	8	0.029	0.31
IPI00022420	Plasma retinol-binding protein	5.76	23029	5	0.024	2.55
IPI00009544	RalA binding protein 1	5.68	76016	2	0.008	1.84
IPI00009544	RalA binding protein 1	5.68	76016	2	0.034	1.73
IPI00019399	Serum Amyloid A-4 protein isoform 1	9.27	14797	2	0.044	0.37
IPI00019399	Serum amyloid A-4 protein isoform 2	9.27	14797	3	0.038	0.58
IPI00550061	Similar to Ig gamma 3 Chain C region	7.47	56823	6	0.032	1.67
IPI00221125	Voltage-gated potassium channel beta-1 subunit	9.23	45462	4	0.006	1.79
IPI00009028	Tetranectin	5.52	22552	7	0.045	1.84
IPI00009028	Tetranectin	5.52	22552	8	0.030	1.68
IPI00022432	Transthyretin	5.52	15877	16	0.022	1.45
IPI00022432	Transthyretin	5.52	15877	7	0.040	3.76
